# Cardiovascular-Related Death Among Women With Breast Cancer in the United States: Do We Need a Better Approach for Estimating Rates?

**DOI:** 10.1177/00333549261477723

**Published:** 2026-08-23

**Authors:** Nguyen Le, Yan Liu, Chanhyun Park

**Affiliations:** 1 Health Outcomes Division, College of Pharmacy, The University of Texas at Austin, Austin, TX, USA; 2 Department of Internal Medicine, Dell Medical School, The University of Texas at Austin, Austin, TX, USA

**Keywords:** breast cancer, cardiovascular, CDC WONDER, SEER, mortality rate

## Abstract

Breast cancer is the most common cancer among women in the United States, and cardiovascular disease (CVD) is a leading cause of death in this population. Understanding CVD-related death among breast cancer survivors is critical to inform policies. Previous studies calculating CVD-related death rates in this population used data from the Centers for Disease Control and Prevention (CDC) Wide-ranging ONline Data for Epidemiologic Research (WONDER) with the general US female population as the denominator, which may have produced misleading comparisons. For example, age-adjusted CVD-related death rates have appeared lower among women with breast cancer than in the general US female population, despite survivors facing an elevated CVD risk, understating the need for CVD-focused survivorship care. We demonstrated 2 methodologically appropriate approaches for estimating CVD-related death rates among US women with breast cancer from 1999 through 2020, using a denominator restricted to the breast cancer survivor population, derived from Surveillance, Epidemiology, and End Results (SEER) and Projected Prevalence (ProjPrev) data. We found trends in CVD-related death rates that were broadly consistent with previous studies, with an average annual percentage change of −1.8 (95% CI, −2.0 to −1.6). Our estimates were more intuitive than those from previous studies because they correctly reflected an elevated, rather than reduced, risk relative to the general population: the age-adjusted CVD-related death rate per 100 000 women with breast cancer was 1078.1 in 1999 and 733.1 in 2020. These findings suggest that our approaches can be applied to future research on cause-specific mortality among patients with cancer using CDC WONDER data. Future studies may improve generalizability and identify strategies for linking databases to generate more accessible evidence for public health decision-making.

## Introduction

Breast cancer is the most common cancer among women in the United States, accounting for approximately 1 in 4 new cancer diagnoses among women each year.^1^ Although survival rates among women with breast cancer have improved substantially,^1,2^ other risks, especially cardiovascular disease (CVD), have emerged as critical in this population.^3-5^ Shared risk factors (eg, obesity)^6^ and cardiotoxic anticancer therapies (eg, anthracyclines)^7,8^ contribute to this elevated CVD risk. CVD-related death has recently been shown to be a critical competing risk, compared with breast cancer–related death, among women with breast cancer.^4,9^ Understanding CVD-related death at the US breast cancer survivor population level is critical to inform policies and services for women with breast cancer.

Several studies have addressed this issue.^10-12^ Although trends in CVD-related death rates among women with breast cancer were the primary focus of these studies, we found that the rate calculations, including crude and age-adjusted rates, were inappropriate because the denominator was misspecified. For example, when age-adjusted to the 2000 US standard population, the CVD-related death rate was significantly higher in the general US female population than among women with breast cancer.^11^ This conclusion was based on using the US female population as the denominator rather than the population of women with breast cancer when estimating the rate. Interpreting these results may not be intuitive for a broader audience because evidence shows that women with breast cancer have a higher risk of CVD-related death than women in the general population.^5^ This substantially lower rate among women with breast cancer may diminish the perceived importance of initiatives for CVD-related death prevention.

The denominator used to calculate the rates did not reflect the population of women with breast cancer but, rather, the US female population. This conventional use of the US female population might be because the Centers for Disease Control and Prevention Wide-ranging ONline Data for Epidemiologic Research (CDC WONDER) database,^13^ which was used by previous studies, only provides denominator data for the US population. By using data from the US population instead of the population with breast cancer as the denominator, CVD-related death rates among women with breast cancer become diluted. We conducted this study to explore this issue.

We first replicated the results of CVD-related death rates among the US female population using CDC WONDER databases. Then, we recalculated the rates using a denominator of women with breast cancer using Surveillance, Epidemiology, and End Results (SEER) databases.^14^

## Methods

### Data Sources and Study Populations

We estimated CVD-related death rates among women with breast cancer from 1999 through 2020 using the CDC WONDER^13^ and SEER databases^14^ with SEER*Stat software version 9.0.42.2 (National Cancer Institute). We derived CVD-related death counts from multiple cause of death data on CDC WONDER for women aged ≥20 years across all racial and ethnic groups, where both diseases of the circulatory system (*International Classification of Diseases, Tenth Revision* [ICD-10] code I00-I99) and breast cancer (ICD-10 code C50) were listed as multiple causes of death.^15^ Because breast cancer is predominant among women compared with men,^1^ we restricted the analysis to women.

We derived estimated prevalence counts of women with breast cancer using limited-duration prevalence statistics provided by SEER*Stat. Detailed information on the statistics using SEER*Stat is available elsewhere.^16^ Briefly, the limited-duration prevalence counts represent the number of people alive on a certain day who were diagnosed with the disease within the past x years (eg, x could be 5 years, 20 years, or other years depending on the data and research questions).^17^ To maximize the number of years when estimating the prevalence, we selected SEER 8 registries, including Connecticut, Hawaii, Iowa, New Mexico, Utah, California (Alameda, Contra Costa, Marin, San Francisco, and San Mateo counties), Georgia (Clayton, Cobb, DeKalb, Fulton, and Gwinnett counties), and Washington (Clallam, Grays Harbor, Island, Jefferson, King, Kitsap, Mason, Pierce, San Juan, Skagit, Snohomish, Thurston, and Whatcom counties).^14^ The prevalence date was set to July 1 of each year, and the maximum available limited-duration prevalence in SEER 8 for each year was used. We then matched the data derived from CDC WONDER with the prevalent data from SEER by states (including counties for several states) and age groups (20-29, 30-39, 40-49, 50-59, 60-69, 70-79, and ≥80 y) to obtain the final data.

Alternatively, the projected prevalence application (ProjPrev) provided by the National Cancer Institute can be used.^18^ The projected prevalence is used to derive the US prevalence of a cancer site of interest by projecting SEER prevalence (using limited-duration prevalence statistics) onto US populations.

### Crude and Age-Adjusted CVD-Related Death Rates

We calculated annual directly age-adjusted CVD-related death rates per 100 000 people for women with breast cancer and the US female population using age adjustment to the 2000 US Census female population distribution.^19^ Specifically, we used the prevalent population of women with breast cancer (limited-duration prevalence derived from SEER 8) as the denominator for rates among women with breast cancer, while the female US population (derived from CDC WONDER) was used as the denominator for rates among the US female population.

### Statistical Analysis

We used Joinpoint version 5.4.0.0 (National Cancer Institute) to assess trends in age-adjusted CVD-related death rates among women with breast cancer by estimating segment-specific annual percentage changes (APCs) and the overall average annual percentage change (AAPC), with first-order autocorrelation (autocorrelation parameter estimated from the data was −0.46). This study was approved by The University of Texas at Austin Institutional Review Board under an exempt protocol.

## Results

**Figure 1.**
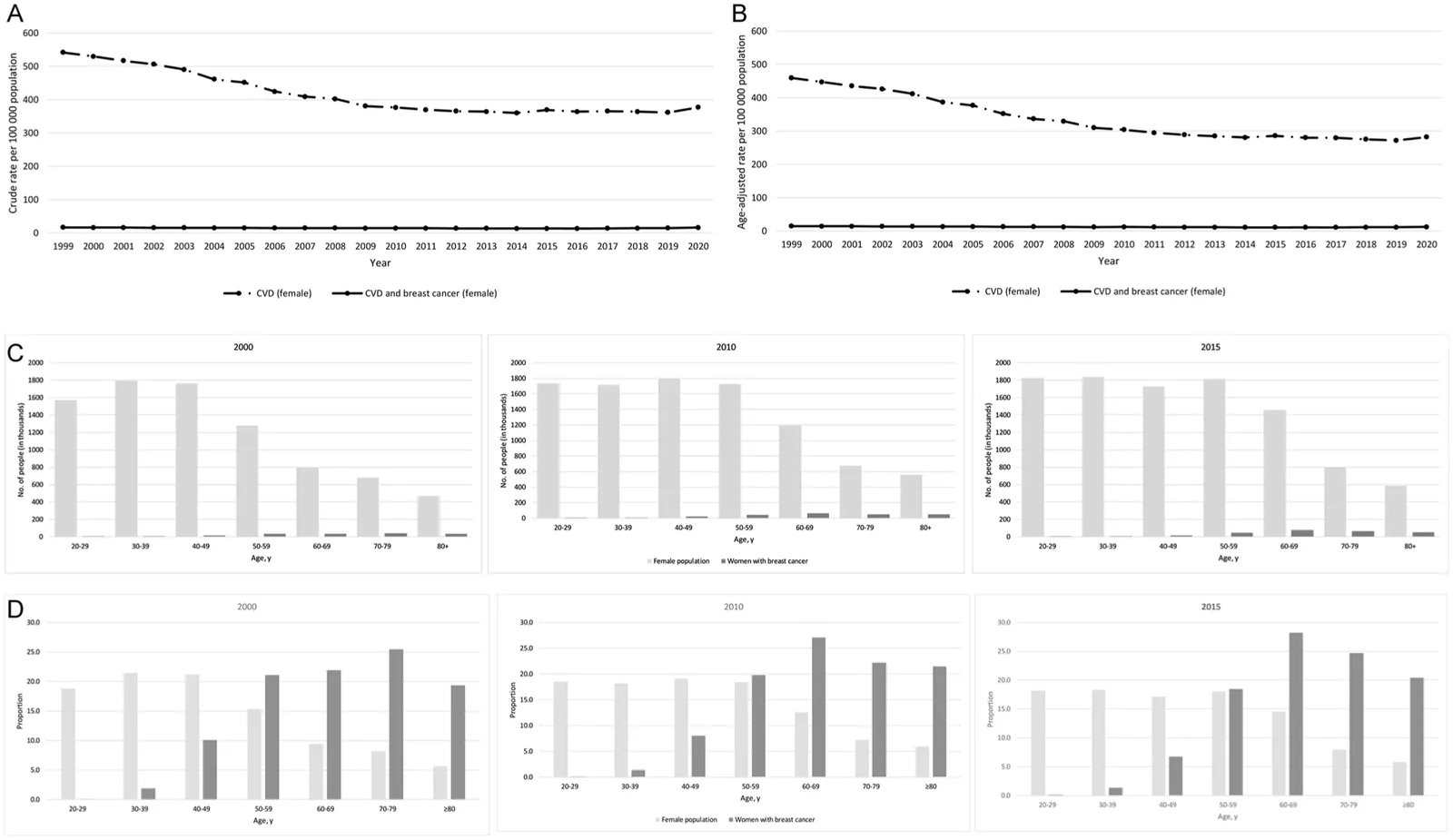
Crude (A) and age-adjusted (B) rates of CVD-related deaths, United States, 1999 through 2020, and age distribution among women aged ≥20 years with breast cancer and the US female population in SEER 8 registry areas in 2000, 2010, and 2015 (C and D). Rates in Panel B are age-adjusted to the 2000 US female population. Data source: Centers for Disease Control and Prevention Wide-ranging ONline Data for Epidemiologic Research.^13^ The 8 areas were Connecticut, Hawaii, Iowa, New Mexico, Utah, California (Alameda, Contra Costa, Marin, San Francisco, and San Mateo counties), Georgia (Clayton, Cobb, DeKalb, Fulton, and Gwinnett counties), and Washington (Clallam, Grays Harbor, Island, Jefferson, King, Kitsap, Mason, Pierce, San Juan, Skagit, Snohomish, Thurston, and Whatcom counties). The prevalence counts of women with breast cancer in each age group were derived from the SEER 8 database.^14^ The prevalence window was set to the maximum available duration (eg, beginning in 1976), and the prevalence date was July 1 of each year. For example, the prevalence count of women with breast cancer in 1999 was the number of women alive on July 1, 1999, diagnosed with breast cancer between 1976 and that date. Abbreviations: CVD, cardiovascular disease; SEER, Surveillance, Epidemiology, and End Results.

We replicated CVD-related deaths among women with breast cancer and the general US female population from 1999 through 2020 using CDC WONDER (Figure 1A and B). The age-adjusted CVD-related death rates, when using CVD-related deaths among women with breast cancer as the numerator and the US female population as the denominator, were 14.4 and 12.3 deaths per 100 000 population in 1999 and 2020, respectively. The age-adjusted CVD-related death rate per 100 000 population among the US female population was 459.4 deaths in 1999 and 282.2 deaths in 2020. Because the US female population was used as the denominator for rate calculations when deriving data from CDC WONDER, we found a substantial gap in rates between the 2 groups.

We examined the distributions of women with breast cancer (derived from SEER 8) and the female population (derived from CDC WONDER) residing in SEER 8 registry areas by age group (Figure 1C and D). The number of women with breast cancer was smaller than the US female population in each age group (Figure 1C). Moreover, the age distribution of women with breast cancer skewed older than the US female population (Figure 1D).

**Figure 2.**
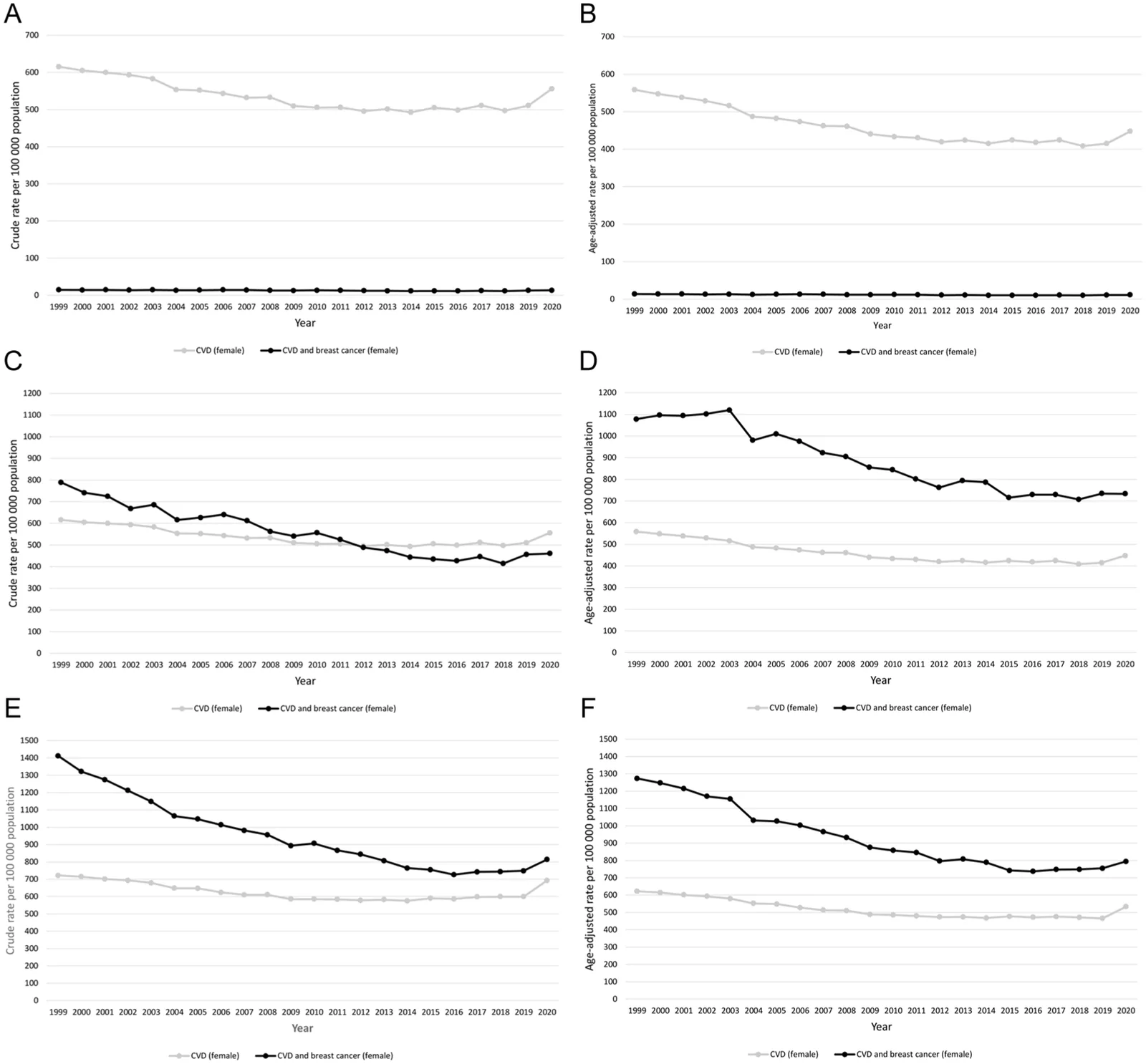
Crude and age-adjusted rates of CVD-related deaths among women with breast cancer and the US female population in SEER 8 registry areas (A-D) and all states (E-F), United States, 1999 through 2020. Panels A and B show crude and age-adjusted (standardized to the 2000 Census US female population) rates using the general US female population as the denominator for both series. Panels C and D show the same rates using the prevalent women with breast cancer population (derived from the SEER 8 database^14^) as the denominator for the breast cancer series (the US female population denominator unchanged for the US series). Panels E and F show the same rates using the projected prevalent women with breast cancer population (derived from the ProjPrev database^18^) as the denominator for the breast cancer series (US female population denominator unchanged for the US series). Abbreviations: CVD, cardiovascular disease; SEER, Surveillance, Epidemiology, and End Results.

We examined the results of CVD-related deaths among women with breast cancer and the general US female population residing in SEER 8 registry areas from 1999 through 2020 (Figure 2A-D). When using the general US female population as the denominator, the rate patterns (Figure 2A and B) were similar to the rates obtained for the entire United States (Figure 1A and B). When the general US female population was used as the denominator for both series, CVD-related death rates among women with breast cancer were substantially lower than those in the general US female population (Figure 2A and B). In contrast, when we used the population of women with breast cancer as the denominator, the crude rates among women with breast cancer were closer to the rates in the general US female population (Figure 2C). The crude CVD-related death rate per 100 000 population among women with breast cancer was 788.9 deaths in 1999 and 593.1 deaths in 2020. Among the general US female population, the crude CVD-related death rates were 615.6 and 555.9 deaths per 100 000 population in 1999 and 2020, respectively. The rates were higher among women with breast cancer than among the general US female population from 1999 to 2011, but the rates were lower among women with breast cancer than among the general US female population from 2012 to 2020.

After age adjustment to the 2000 US population, the CVD-related death rate among the general US female population changed modestly, while the rate among women with breast cancer increased in all years (Figure 2D). Rates per 100 000 population among the general US female population were 558.5 deaths and 447.5 deaths in 1999 and 2020, respectively. The CVD-related death rate per 100 000 population among women with breast cancer was 1078.1 deaths in 1999 and 733.1 deaths in 2020. We obtained similar results when estimating CVD-related death rates among women with breast cancer in the United States (without being restricted to only 8 states) using the ProjPrev to derive the projected prevalence of women with breast cancer (Figure 2E and F).

Joinpoint regression analysis showed findings consistent with previous studies, despite being based on SEER 8 registry areas. Overall, CVD-related death rates among women with breast cancer decreased from 1999 to 2020, with an AAPC of −1.8 (95% CI, −2.0 to −1.6). Rates decreased during 2002-2011 (APC = −3.5; 95% CI, −4.8 to −3.1) and 2011-2017 (APC = −1.9; 95% CI, −3.5 to −1.1). Rates were stable during 1999-2002 (APC = 1.0; 95% CI, −0.9 to 4.6) and 2017-2020 (APC = 0.9; 95% CI, −1.0 to 3.6).

## Discussion

We demonstrated 2 approaches that are methodologically more appropriate than those used in previous studies^10-12^ for calculating the CVD-related death rate among women with breast cancer using CDC WONDER and SEER databases. Disparities in age distributions across the 2 groups suggest the importance of using the population of women with breast cancer as the denominator when calculating rates and adjusting rates to the US population. Although our trends were aligned with previous studies,^10-12^ the rates were more intuitive for a broader audience. We acknowledge several limitations. First, because long-term follow-up data on the population of women with breast cancer for the entire United States are limited, the rates only represent data for SEER 8 registry areas and are not generalizable to the US population of women with breast cancer. There are several available data sources for the US breast cancer survivor population, such as the North American Association of Central Cancer Registries (NAACCR) products, although the follow-up duration is shorter than SEER 8. This limitation might be mitigated when more generalizable data with longer follow-up become available.^20^ Second, using limited-duration prevalence statistics may need to assume there was no inbound migration of cancer survivors from other states who would die in the SEER states of interest; however, the ProjPrev^18^ can be used to mitigate this limitation. Third, evidence indicates racial disparities in CVD-related deaths among women with breast cancer^21,22^; thus, race may need to be accounted for in addition to age. However, we could not adjust for race in our analysis because of limited data. Future studies may increase the data size by using larger SEER registries (eg, SEER 12, SEER 17, SEER 22), although doing so may shorten the available study period depending on registry coverage. Finally, we did not address whether to standardize rates to the 2000 US female population or the population of women with breast cancer, a critical question raised by Watkins et al.^23^ We believe that adjusting to the 2000 US female population may suffice and is more meaningful because it allows comparisons of the rates with the general population and across cancer types.

## Conclusion

These approaches yielded estimates that more accurately reflect the elevated CVD risk in this population and can be applied to future research on cause-specific mortality in other patients with cancer. Future studies should test the generalizability of these methods to other cancer populations, explore strategies for linking databases to generate more accessible evidence, and examine how such corrected estimates could inform cardio-oncology guidelines and public health resource allocation.
